# Supplementary material for: Encapsulate α-MnO2 nanofiber within graphene layer to tune surface electronic structure for efficient ozone decomposition
Source: Nat Commun. 2021 Jul 6;12:4152. doi: 10.1038/s41467-021-24424-x (PMC8260790; doi:10.1038/s41467-021-24424-x)
Supplement: Supplementary file 1 — Supplementary Information [file 41467_2021_24424_MOESM1_ESM.pdf]

Supporting Information

Encapsulate α-MnO_2 nanofiber within graphene layer to tune surface electronic structure for efficient ozone decomposition

Guoxiang Zhu,[a,b] Wei Zhu,[a,c] Yang Lou,[d] Jun Ma,[a,e] Wenqing Yao,[a] Ruilong Zong,[a] Yongfa Zhu[a]*

[a]Department of Chemistry, Tsinghua University, Beijing 100084, China

[b]Institute of Chemical Materials, China Academy of Engineering Physics, Mianyang Sichuan, 621900, China

[c]College of Environmental and Chemical Engineering, Xi'an Polytechnic University, Xi'an 710048, China

[d]Key Laboratory of Synthetic and Biological Colloids, Ministry of Education, School of Chemical and Material Engineering, Jiangnan University, Wuxi Jiangsu, 214122, China

[e]College of Chemistry and Materials Science, Sichuan normal university, Chengdu Sichuan, 610066, China

Corresponding author:

Yongfa Zhu

Address: Department of Chemistry, Tsinghua University, Beijing, 100084, China;

Email: zhuyf@mail.tsinghua.edu.cn;

Tel.: (+86)10-6278-7601;

Fax: (+86)10-6278-7601;

Contents

Section A. Supplemental Methods

Section B. Supplemental Data

Figure S1. The structure parameters of synthesized 7.50% MnO_2@GR catalysts.

Figure S2. HR-TEM images of 7.50% MnO_2@GR at different regions.

Figure S3. HAADF-STEM image (a) and corresponding EDX linear scanning (b) and maps scanning maps of α-MnO_2 for K (d), O (e), Mn (f) and combined image (c).

Figure S4. Optical micrographs of α-MnO_2 (a) and 7.50% MnO_2@GR (b) with 100 magnifications.

Figure S5. The structure parameters of synthesized MnO_2@GR catalysts.

Figure S6. The morphology of synthesized MnO_2@GR catalysts.

Figure S7. The morphology of synthesized MnO_2@GR catalysts.

Figure S8. O 1s spectra of fresh α-MnO_2 nanowire and 7.50% MnO_2@GR.

Figure S9. The catalytic performance and structural parameters of α-MnO_2, GO/MnO_2 and GO+MnO_2 catalysts.

Figure S10. SEM images of GR (a), α-MnO_2 (b), GO/MnO_2 (c) and 7.5% MnO_2@GR (d).

Figure S11. The effect of graphene layer on the ozone conversion of 7.50% MnO_2@GR calcinated at 350°C for 4 h under air atmosphere.

Figure S12. The performance of regenerated α-MnO_2 nanowires at different relative humidity.

Figure S13. The regeneration ability of MnO_2@GR catalysts under different conditions.

Figure S14. The XRD patterns of OMS-2-HH and MnO_x-HHB catalysts.

Figure S15. TEM images of OMS-2-HH (a) and MnO_x-HHB (b). HRTEM images of OMS-2-HH (c) and MnO_x-HHB (d).

Figure S16. The comparison on the regeneration ability of MnO_2@GR and reported catalysts under different conditions.

Figure S17. SEM images of 7.50% MnO_2@GR coated stainless steel mesh.

Figure S18. The accumulation of intermediated oxygen species.

Figure S19. Analysis of the surface bonding environment via XPS.

Figure S20. The AOS of Mn, surface adsorbed oxygen content in O 1s after 7.50% MnO_2@GR treated with ozone for different time.

Figure S21. Dissociative chemisorption of an ozone molecule from the physisorbed states.

Figure S22. The simulated structures of MnO_2 (a) and graphene MnO_2-one-OV/GR and MnO_2-ten-OV/GR. The initial crystal structures of MnO_2 (a) and graphene. (b) The models of MnO_2-one-OV/GR and MnO_2-ten-OV/GR heterojunctions.

Figure S23. The optimized structure of MnO_2-ten-OV@GR with different graphene layers and their charge density differences.

Figure S24. Ultraviolet Photoelectron Spectroscopy of Au.

Figure S25. Surface potential and UPS spectra of α-MnO_2, 7.50% MnO_2@GR and GR.

Figure S26. The front view of the charge density differences (above) in MnO_2-one-OV@GR (a) and MnO_2-ten-OV@GR (b)

Figure S27. The model of graphene unit (left) and ozone molecule (right).

● Section A: Supplemental Methods

A1: Catalyst characterization.

X-ray diffraction (XRD) patterns were collected via X-ray diffractometer (Rigaku D/max-2400, λ = 1. 5406Å). Morphologies of the samples were obtained by a Field Emission Gun Scanning Electron Microscopy (FESEM, Hitachi SU-8010) and a transmission electron microscopy (TEM, Hitachi 7700) with an accelerating voltage of 100 kV. High resolution transmission electron microscopy (HRTEM) images were captured via a JEM 2100F field emission transmission electron microscope at an accelerating voltage of 200 kV. The element composition and distribution were recorded by an energy dispersive (EDS) detector equipped in JEM 2100F. XPS spectra were was conducted in a PHI Quantera SXM^{TM} system and the binding energy was calibrated with the signal for adventitious carbon at 284.8 eV. FT-IR spectra was recorded by Bruker V70 spectrometer. CHI-660D electrochemical system was used to examine the electrochemical measurements. Electrochemical impedance spectroscopy (EIS) were measured in a three-electrode quartz cells using 0.1 M Na_2SO_4 as electrolyte solution. SCE served as reference electrode, platinum wire served as counter electrode, and sample film electrodes on glassy-carbon electrode used as working electrode.

Temperature programmed desorption (TPD) was carried out to on a Cat-Lab (BEL Japen, Inc.) equipped with an online QIC-200 quadrupole mass (Inprocess Instruments, GAM 200) as a detector. Firstly, 20 mg sample was added into the U-type quartz reactor and heated at 120°C for 2 h in Ar atmosphere. When it cool to the room temperature, the sample was kept in a wet gas flow (50°C, Ar, RH=70%) for 1 h. Then the TPD process was carried out with a heating rate of 5°C/min.

A2: Catalyst evaluation.

The performance of catalyst for ozone decomposition was evaluated in a continuous fixed bed reactor at 25ºC. For each test, 100 mg catalyst was used and the gas flowrate into the reactor was maintained at 900 mL/min. Ozone was generated by arc discharge in O_2 stream and the inlet ozone concentration was kept at 50 ± 1 ppm by tuning the discharge voltage and the gas

flowrate through the ozonator (model 1000BT-12, Shanghai Enaly Mechanical and Electrical Technology Company). Then, the generated ozone mixed adequately with clear air in a mixing drum and then transported into the reactor. The inlet and outlet ozone concentration was recorded (model 202, 2B Technologies) and the ozone conversion was calculated through the following equation:

$$\text{Ozone removal rate} = 100\% \times (C_{in} - C_{out})/C_{in}. \quad (1)$$

where C_{in} and C_{out} present inlet and outlet ozone concentration respectively.

A3: DFT calculation methods

All calculation were accomplished using density functional theory (DFT) with the projected augmented wave (PAW) method, as implemented within the VASP code. The Perdew-Burke-Ernzerhof (PBE) with Hubbard U corrections of generalized gradient approximation (GGA + U) was utilized as the exchange-correlation functional. In this work, we employ values of U-J = 3.9 eV, for the spherical part of the interaction, and J = 1.0 eV.[1] The valence atomic configurations were Mn: $3p^6 3d^5 4s^2$, O: $2s^2 2p^4$, C: $2s^2 2p^3$, respectively. The energy cutoff for a plane wave basis set was 400 eV, and a gamma centered Monkhorst-Pack k-mesh of 2 × 2 × 1 was used for heterojunction calculations. In geometry optimizations, the self-consistent convergence accuracy was set at 1×10^{-6} eV/atom and the atomic Hellman-Feynman forces are smaller than 0.02eV/Å. Furthermore, spin polarization, vdW correction proposed by Grimme and Dipole correction along Z direction were considered in all calculations.

The tetragonal MnO_2 with space group of I 4/M was selected as bulk MnO_2, in which each cell contained 36 atoms (12 Mn atoms and 24 O atoms). The lattice parameters of MnO_2 are a= 9.92Å, b= 9.92Å, c= 2.93Å, α= β= γ=90°. For bulk graphene, the hexagonal graphene with P63/mmc space group was taken, each cell contained 10 atoms and the lattice parameters of graphene are a= b= 2.47Å, c= 8.69Å, α= β=90°, γ=120°. **Figure S1a, b** shows the initial crystal structures of MnO_2 and graphene. Here, MnO_2-one-OV/graphene and MnO_2-ten-OVs/graphene heterojunctions were modeled as the supercell slab, in which the geometry structure contained parallel graphene (001) and MnO_2 (110) sheet. The (110) crystal plane of

MnO_2 was considered, because it has the lowest surface energy and is easy to dominate the largest surface area comparing with other crystal planes.[2] A commensurability was taken between graphene and MnO_2 sheet, where a 5×1 lateral periodicity of MnO_2 and 6×7 lateral periodicity of graphene sheet were employed. A vacuum space of 15Å in the Z direction was adopted to isolate slab as boundary condition. The side view of geometric structures of MnO_2-one-OV/graphene and MnO_2-all-OVs/graphene heterojunctions were shown in **Figure S1c, d**. The initial lattice parameters between the optimized MnO_2 and graphene sheet were presented in **Table S1**. The lattice mismatch rations of initial lattice parameters are 1.20% for a and 4.43% for b, respectively.

- **Section B: Supplemental Data**

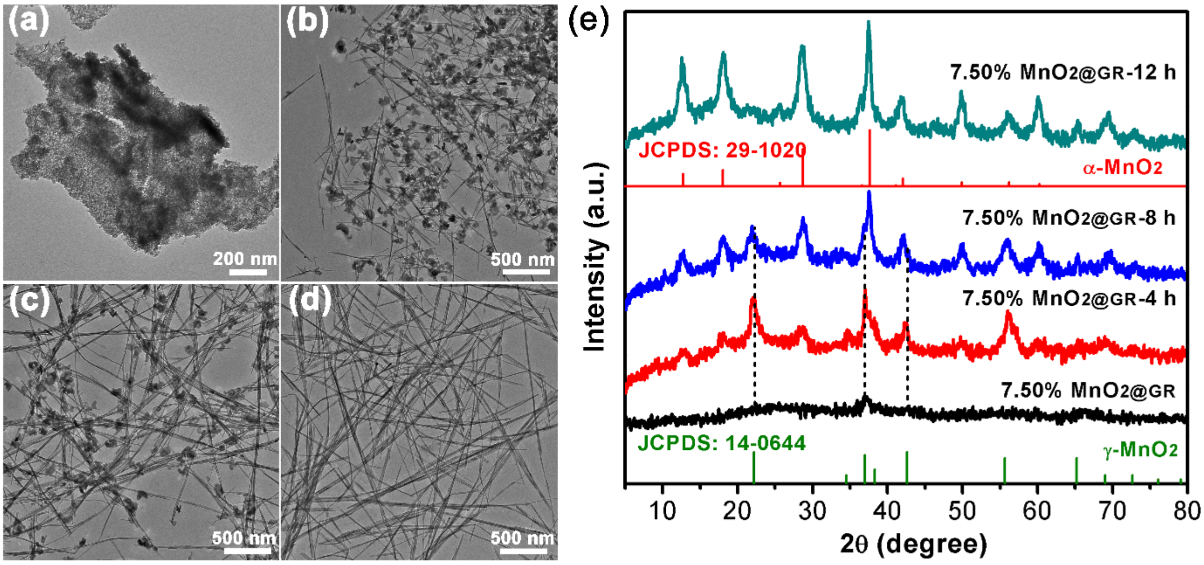

Figure S1. The structure parameters of synthesized 7.50% MnO_2@GR catalysts. (a-d) TEM images of 7.50% MnO_2@GR with varied hydrothermal time of 0 h, 4 h, 8 h, 12 h respectively. (e) XRD patterns of 7.50% MnO_2@GR with varied hydrothermal time.

Ultrathin graphene encapsulated α-MnO_2 nanofibers were prepared by a "complexation-reaction-growth" procedure.[3] Amorphous MnO_2 firstly formed on the graphene, then MnO_6 octahedron units grew into α-MnO_2 and further transferred into α-MnO_2 nanofiber. At the same times, large-area graphene was further exfoliated and frizzled, finally displaying a core-shell structure. When the hydrothermal time kept at 12 h, the obtained sample displayed a pure α-MnO_2 (JCPDS No. 29-1020), in line with the variation of the morphology.

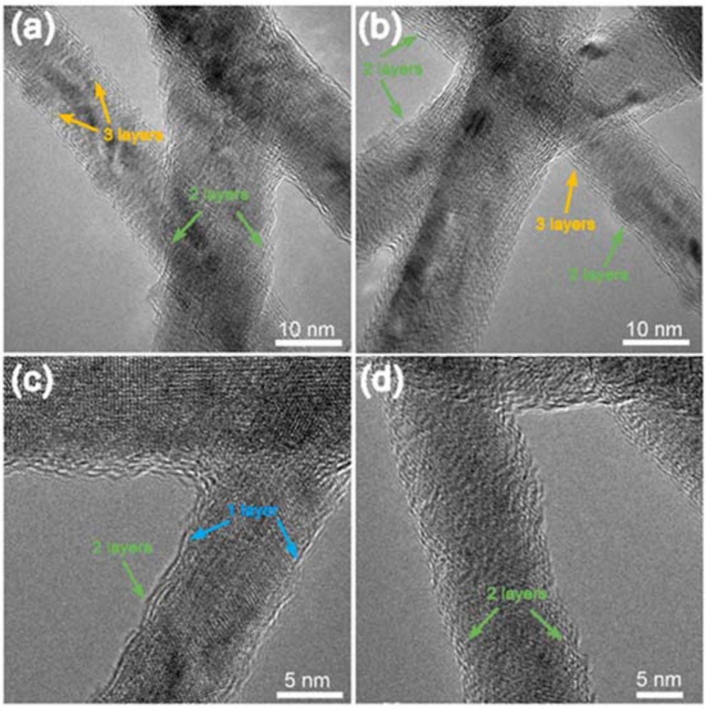

Figure S2. HRTEM images of 7.50% MnO_2@GR at different regions.

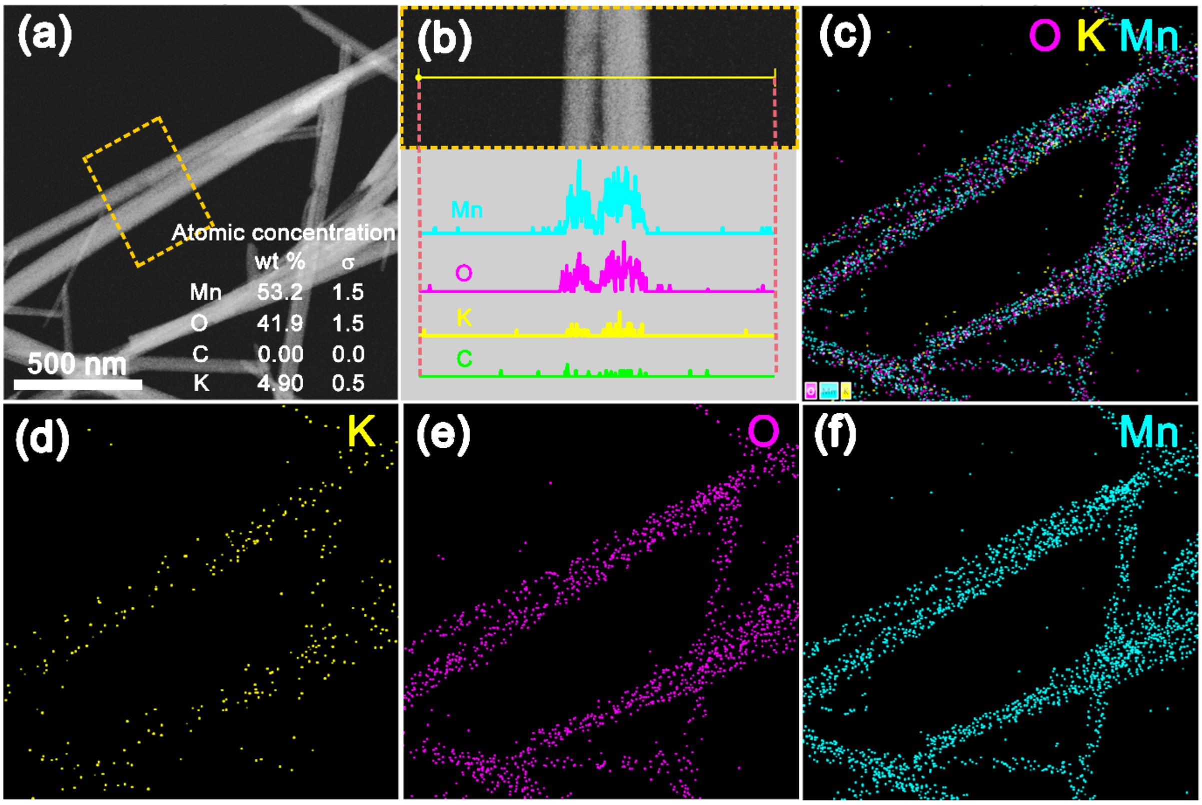

Figure S3. HAADF-STEM image (a) and corresponding EDX linear scanning (b) and maps scanning maps of α-MnO_2 for K (d), O (e), Mn (f) and combined image (c).

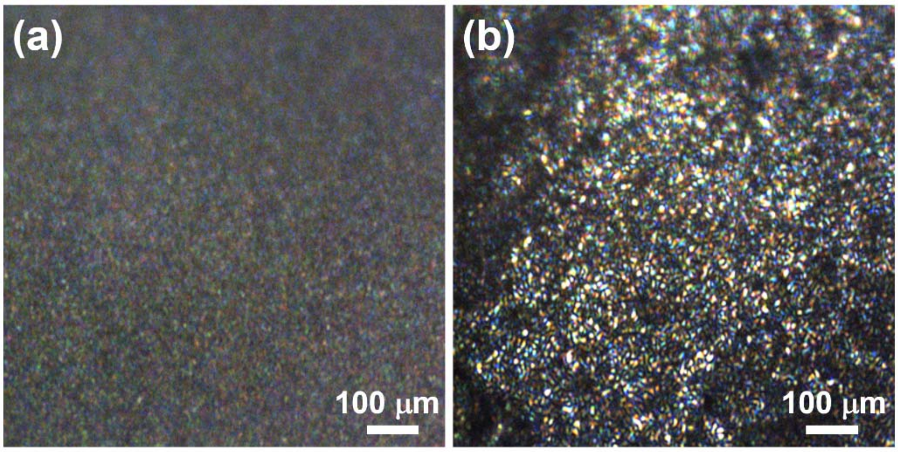

Figure S4. Optical micrographs of α-MnO_2 (a) and 7.50% MnO_2@GR (b) with 100 magnifications.

The optical micrographs shown that 7.50% MnO_2@GR presented an obvious high gloss, which often appeared in graphene. This indicated that 7.50% MnO_2@GR presented a uniform core-shell structure rather than local phenomenon. Therefore, the exposed surface in 7.50% MnO_2@GR almost was graphene shells.

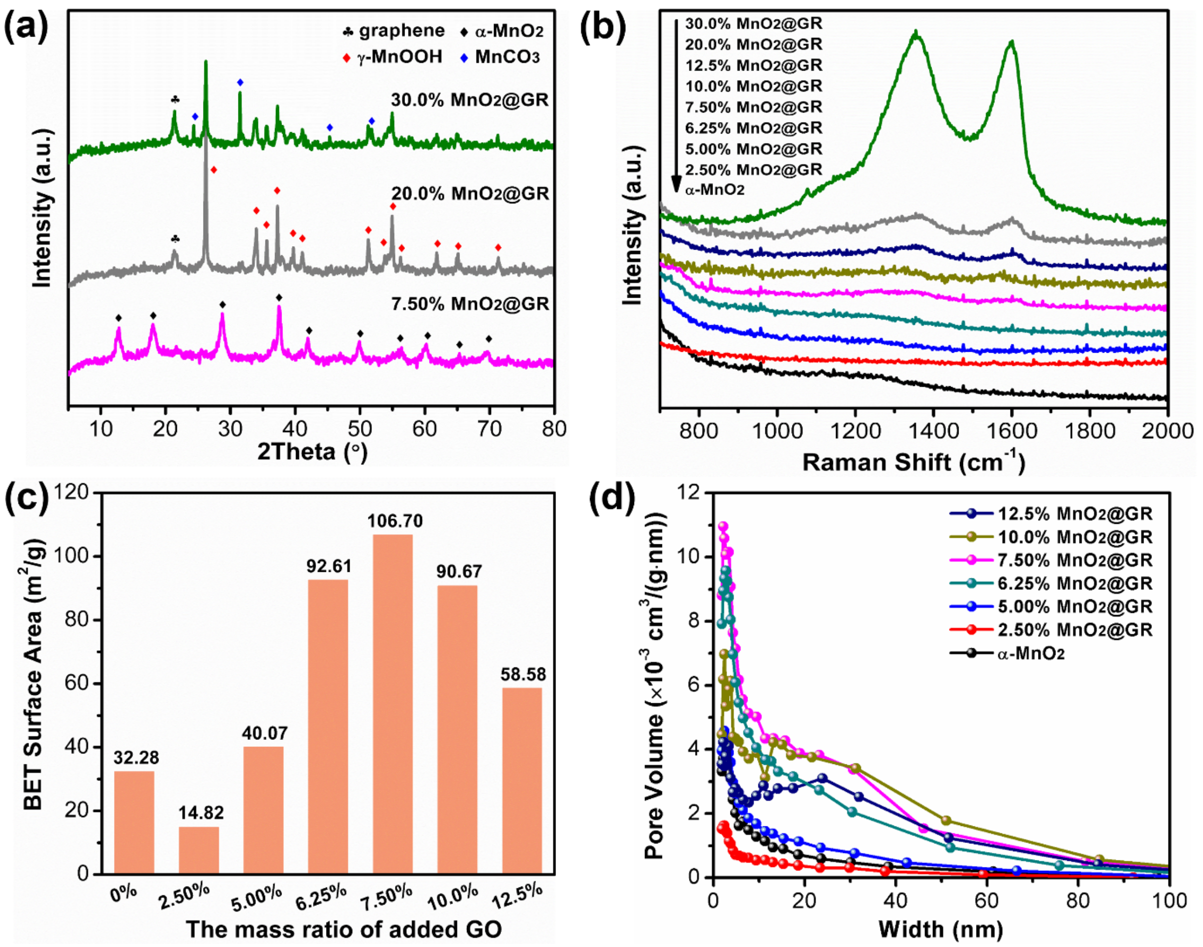

Figure S5. The structure parameters of synthesized MnO_2@GR catalysts. (a) XRD patterns of 7.50% MnO_2@GR, 20.0% MnO_2@GR, 30.0% MnO_2@GR. Raman shift (b), BET surface areas (c) and pore size distribution (d) of MnO_2@GR with different ratio of GR.

As shown in **figure S5a**, α-MnO_2 would transfer to α-MnOOH and $MnCO_3$, further confirming that the crystal structure of manganese oxide varied with the increase of the GO content, in agreement with the latest report.[4] Besides, the diffraction peak of graphene was only found in the XRD patterns of 20.0% MnO_2@GR, 30.0% MnO_2@GR, indicating a low content of graphene in 7.50% MnO_2@GR. The Raman spectra (**figure S5b**) shown the peak intensity at 1347 cm^{-1} and 1610 cm^{-1} increased with the increase of the GO content, confirming the ultrathin graphene shells indeed existed on the surface of 7.50% MnO_2@GR. The BET surface areas of 7.50% MnO_2@GR also increased from 32.28 to 106.70 m^2/g (**figure S5c**), comparing with MnO_2, offering abundant active sites for ozone decomposition. The pore size distribution (**figure S5d**) shown a large amount of micropore and mesoporous existed in 7.50% MnO_2@GR, corresponding to its hierarchical structure.

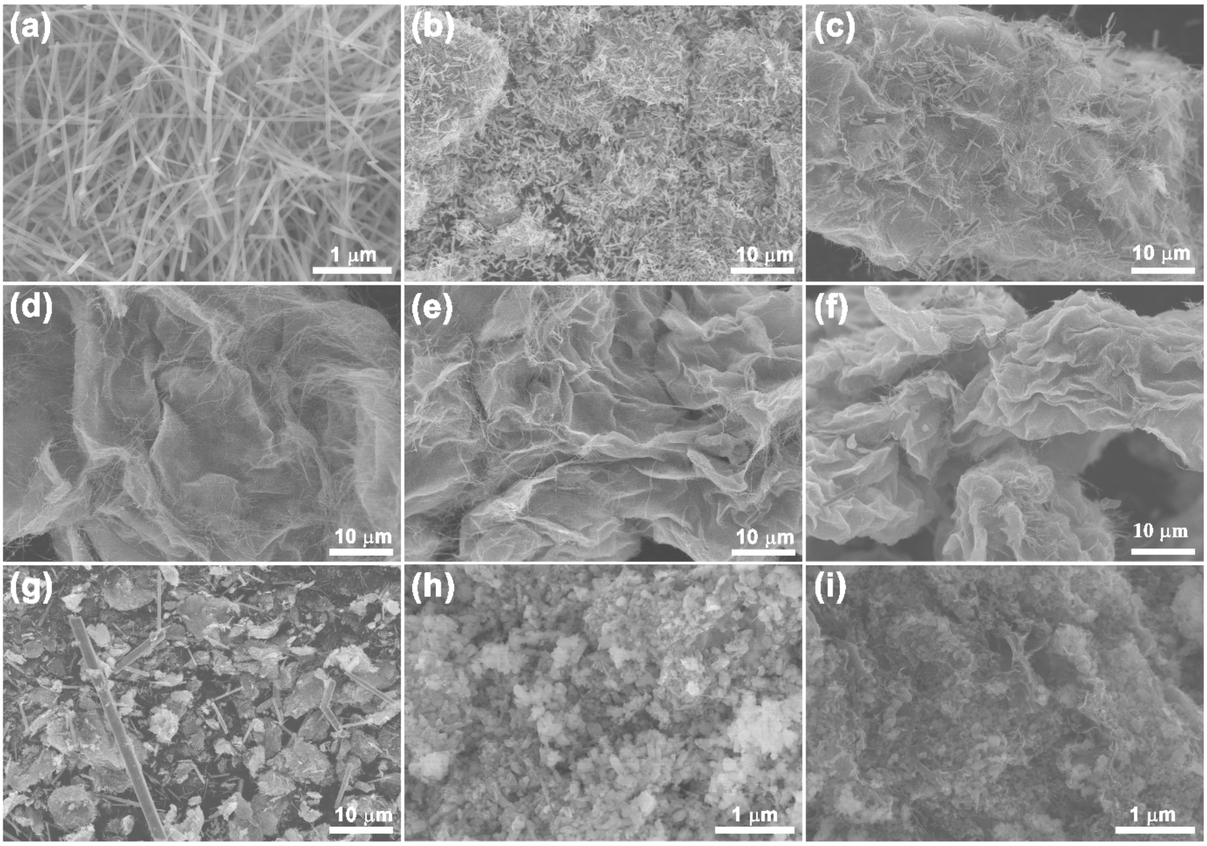

Figure S6. The morphology of synthesized MnO_2@GR catalysts. SEM images of α-MnO_2 nanowire (a), 2.50% MnO_2@GR (b), 5.00% MnO_2@GR (c), 6.25% MnO_2@GR (d), 7.50% MnO_2@GR (e), 10.0% MnO_2@GR (f), 12.5% MnO_2@GR (g), 20.0% MnO_2@GR (h) and 30.0% MnO_2@GR (i).

As shown in **figure S6**, the morphology varied with GO content, corresponding to the changes of the diffraction peak in XRD pattern. When GO content was lower than 7.5%, short nanorod was found, corresponding to α-MnO_2. When GO content reached at 7.5%, α-MnO_2 totally transferred to α-MnO_2 nanofiber. If GO content increased continuously, α-MnOOH and $MnCO_3$ appeared. As shown in **figure S6e**, 7.50% MnO_2@GR displayed a lamellar structure, similar to graphene network structure. Thus, it can be concluded that α-MnO_2 nanofiber growth on the graphene layer.

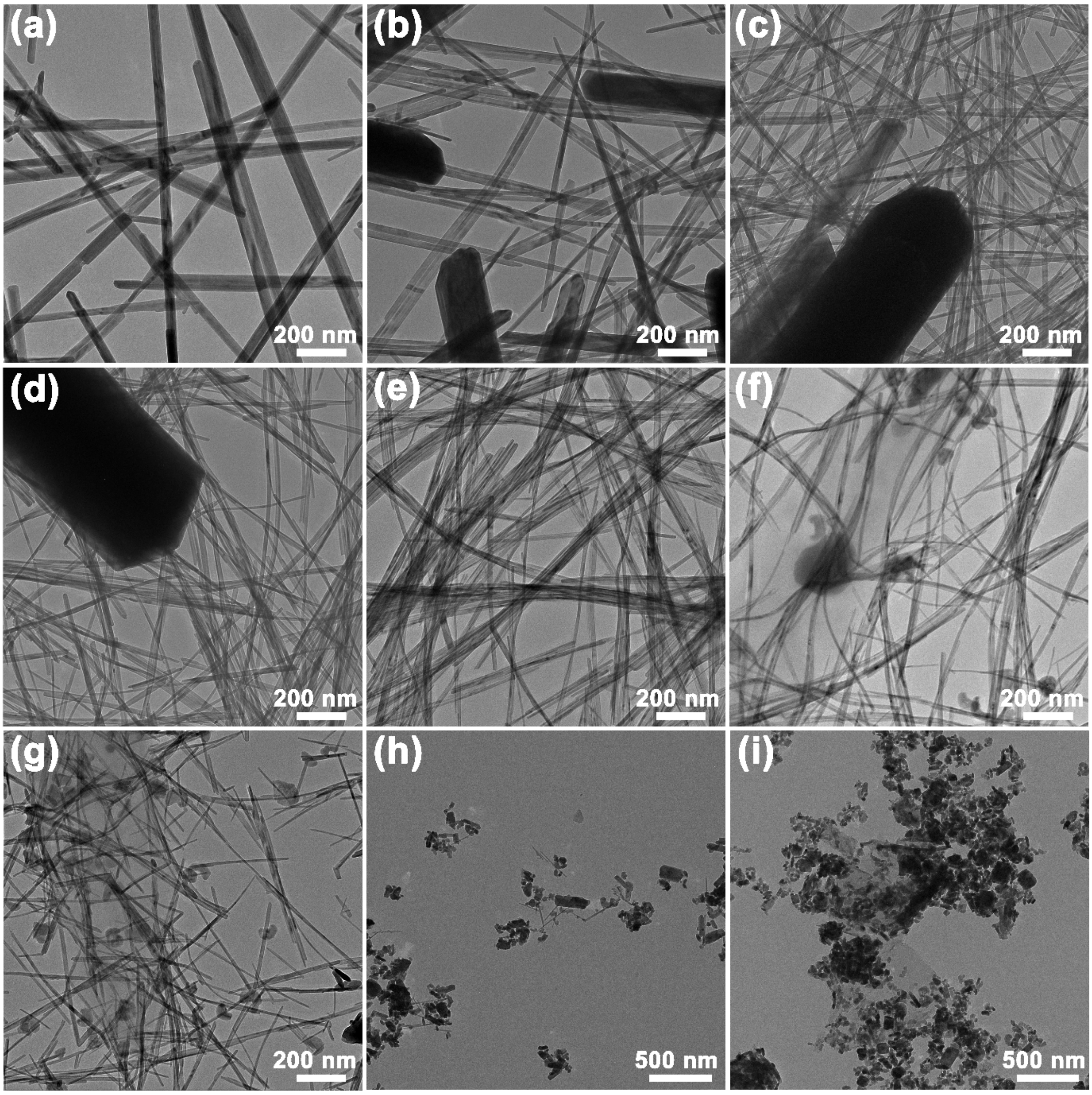

Figure S7. The morphology of synthesized MnO_2@GR catalysts. TEM images of EM i_2 nanowire (a), 2.50% MnO_2@GR (b), 5.00% MnO_2@GR (c), 6.25% MnO_2@GR (d), 7.50% MnO_2@GR (e), 10.0% MnO_2@GR (f), 12.5% MnO_2@GR (g), 20.0% MnO_2@GR (h) and 30.0% MnO_2@GR (i).

As shown in **figure S7**, the effect of GO content on the crystal structure and morphology was further confirmed by TEM images. Importantly, graphene layer was not found until GO content reached at 10.0%, confirming that added GO almost coated on the α-MnO_2 nanofiber. According to SEM and TEM images, it can be concluded that graphene layer was destroyed and coated on the surface of α-MnO_2 nanofiber under the process of crystal reconstruction, consequently displaying a core-shell structure.

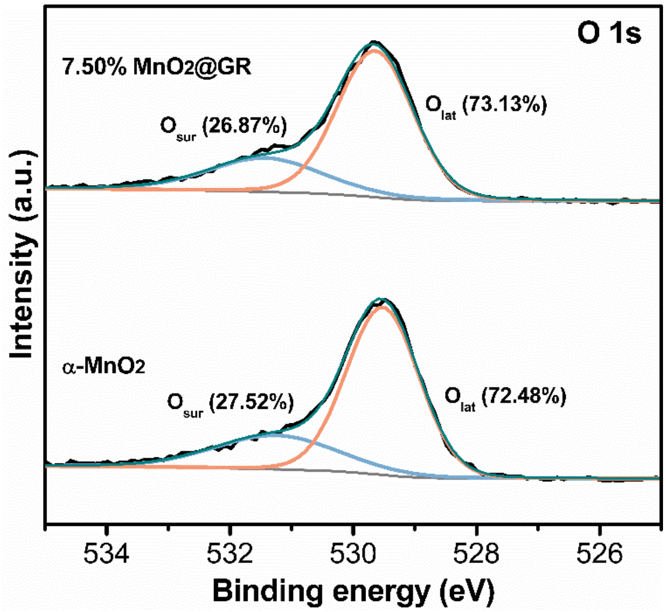

Figure S8. O 1s spectra of fresh α-MnO_2 nanowire and 7.50% MnO_2@GR.

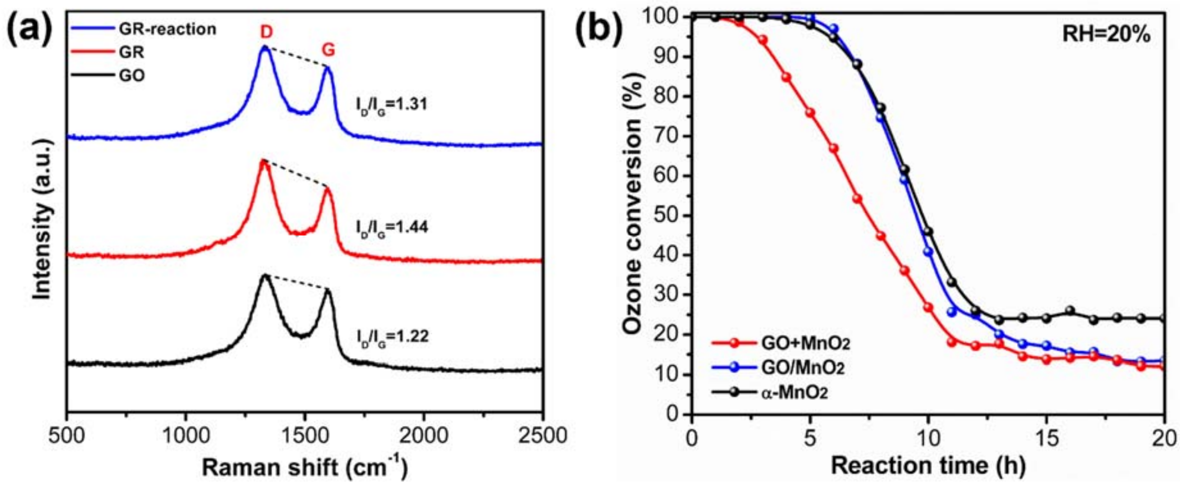

Figure S9. The catalytic performance and structural parameters of α-MnO_2, GO/MnO_2 and GO+MnO_2 catalysts. (a) Raman shift of GO, GR, GR-reaction (reaction for 20 h). (b) Ozone conversion on α-MnO_2, GO/MnO_2 and GO+MnO_2 (physical mixture).

1.0 g obtained α-MnO_2 nanowires physically mixed with 75 mg GO aerogel (dehydrated via a freeze-drying process) to prepare GO+MnO_2. In the Raman spectra, the peaks located at 1334 and 1597 cm^{-1}, which were assigned to the G band and D band of graphene respectively and were used to quantify the density of defects in sp^2 carbon atoms.[5] **Figure S9a** shown the integrated intensity ratio of I_D/I_G increased from 1.22 to 1.44 after a hydrothermal reduction, indicating more nongraphitic impurities formed in GR. After treated in ozone for 20 h, the ratio of I_D/I_G decreased to 1.31, suggesting the nongraphitic impurities could react with ozone molecule.

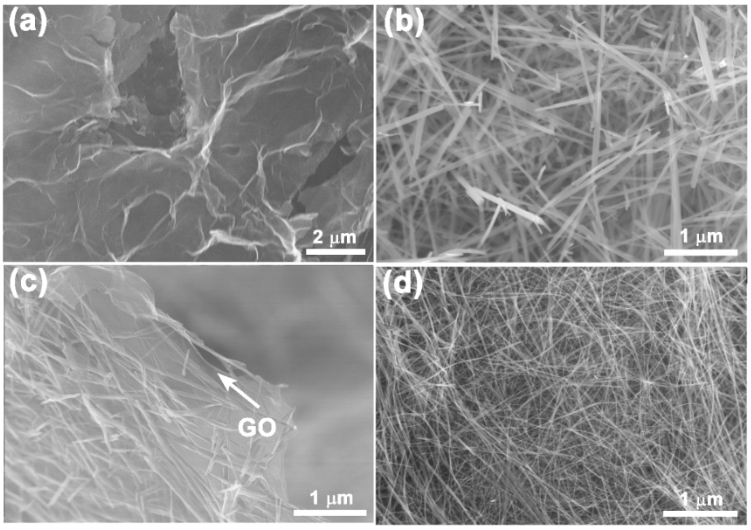

Figure S10 SEM images of GR (a), α-MnO_2 (b), GO/MnO_2 (c) and 7.5% MnO_2@GR (d).

FESEM images in **figure S10** showed that GO/MnO_2 presented a regular composite structure, in which α-MnO_2 nanowires were located on the surface of the large-areas graphene.

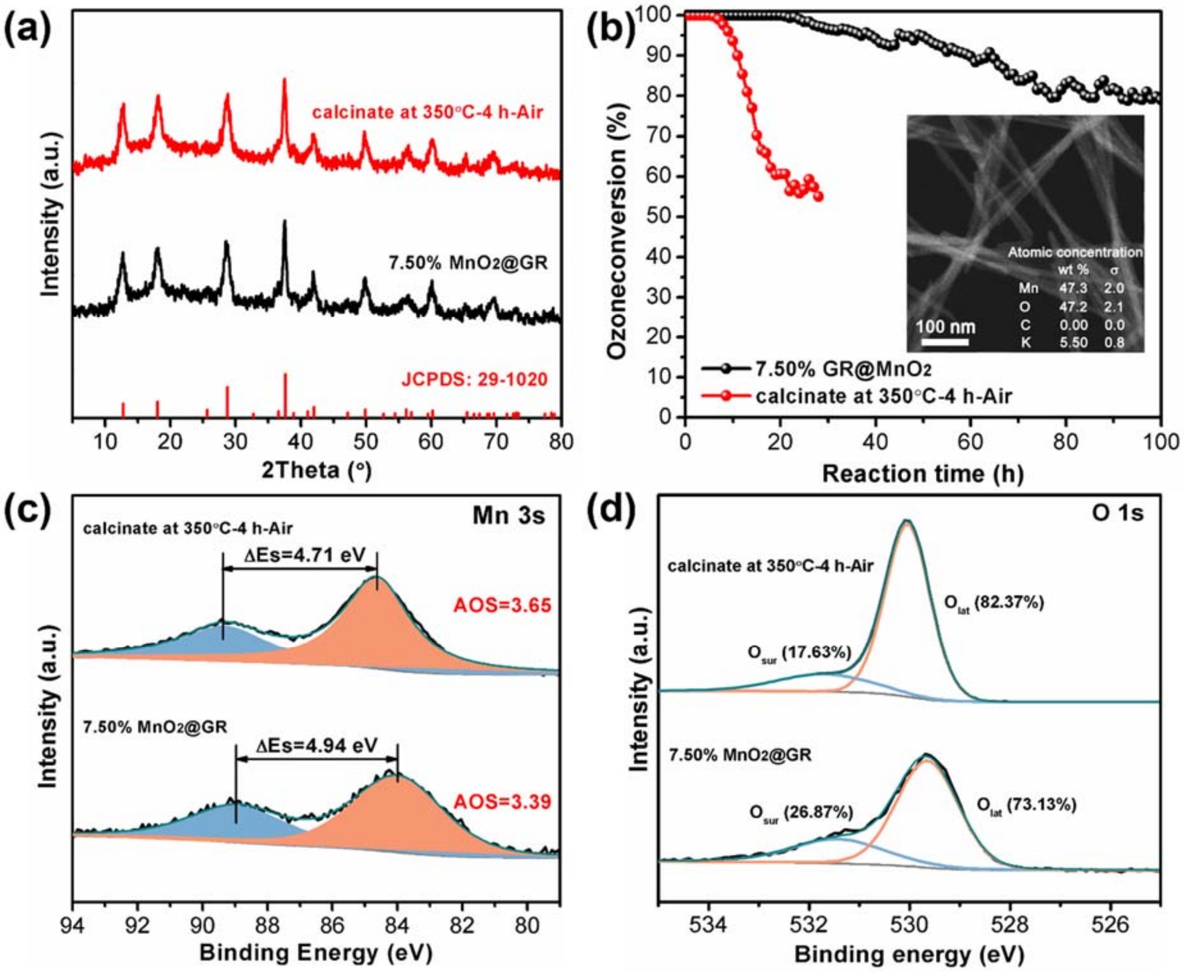

Figure S11. The effect of graphene layer on the ozone conversion of 7.50% MnO_2@GR calcinated at 350ºC for 4 h under air atmosphere. (a) XRD patterns of 7.50% MnO_2@GR before and after calcination. (b) Ozone conversion of 7.50% MnO_2@GR before and after calcination and the element content for calcinated 7.50% MnO_2@GR obtained by EDS map scanning. Mn 3s (c) and O1s (d) spectra of 7.50% MnO_2@GR before and after calcination. Experimental conditions: 0.1 g catalyst, 50 ppm O_3, flow rate = 900 mL/min, RH=20%, 25°C.

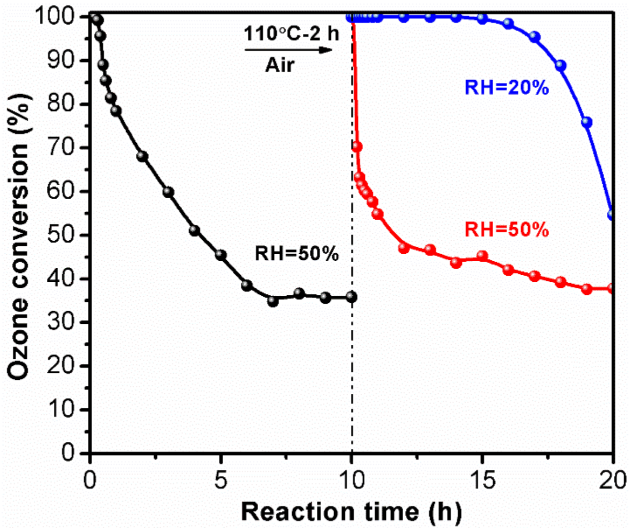

Figure S12. The performance of regenerated α-MnO_2 nanowires at different relative humidity.

As shown in **figure S12**, the regenerated α-MnO_2 nanowires exhibited an almost the same ozone conversion of fresh α-MnO_2 nanowires at 20% RH, indicating that the decline of the ozone conversion at 50% RH resulted from water adsorption rather than the accumulation of the intermediated oxygen species.

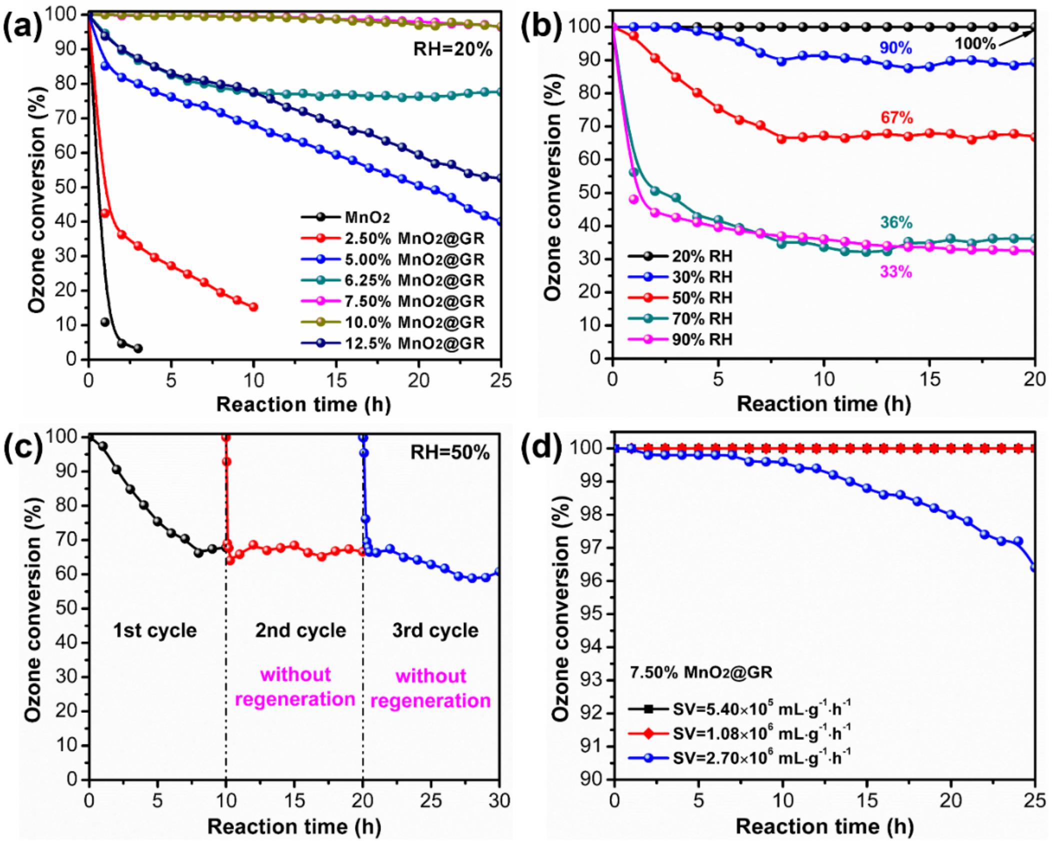

Figure S13. The regeneration ability of MnO_2@GR catalysts under different conditions. (a) Ozone conversion on MnO_2@GR with different content of GR (20 mg catalyst was used). (b) Ozone conversion on 7.50% MnO_2@GR at different relative humidity. (c) The cycle performance of 7.50% MnO_2@GR at 50% RH. (d) Ozone conversion on 7.50% MnO_2@GR at different space velocity. Experimental conditions: 0.1 g catalyst (except for (a) and (d)), 50 ppm O_3, flow rate = 900 mL/min, 25 °C.

Above results have indicated that the GO addition has a significant effect on the morphology and crystal structure of the catalyst. As displayed in **figure S13a**, 7.50% MnO_2@GR exhibited the highest ozone conversion among the samples with different GR content, suggesting that the unique core-shell structure is important for the ozone decomposition. In addition, we investigated the ozone conversion on 7.50% MnO_2@GR under different RH, as displayed in **figure S13b**. Because of the competitive adsorption, the conversion shown a sharp decrease and then remained stable in the wet gas flow. Although the stable conversion decreased with the increase of the RH, 7.50% MnO_2@GR remained 67% ozone conversion at 50% RH, significantly higher than the ratio (35%, **figure 4b in the main manuscript**) of α-MnO_2 nanowire. This suggested that the graphene indeed enhanced the ozone conversion efficiency under high humidity. As shown in **figure S13c**, the cycle performance of 7.50% MnO_2@GR

was evaluated at 50% RH to explain the effect of regeneration. **figure S13d** has shown the ozone conversion on 7.50% MnO_2@GR under different mass space velocity (WHSV). This indicated that the real catalytic performance of 7.50% MnO_2@GR can be reflected when the space velocity is below 1.80×10^6 $mL\cdot g^{-1}\cdot h^{-1}$.

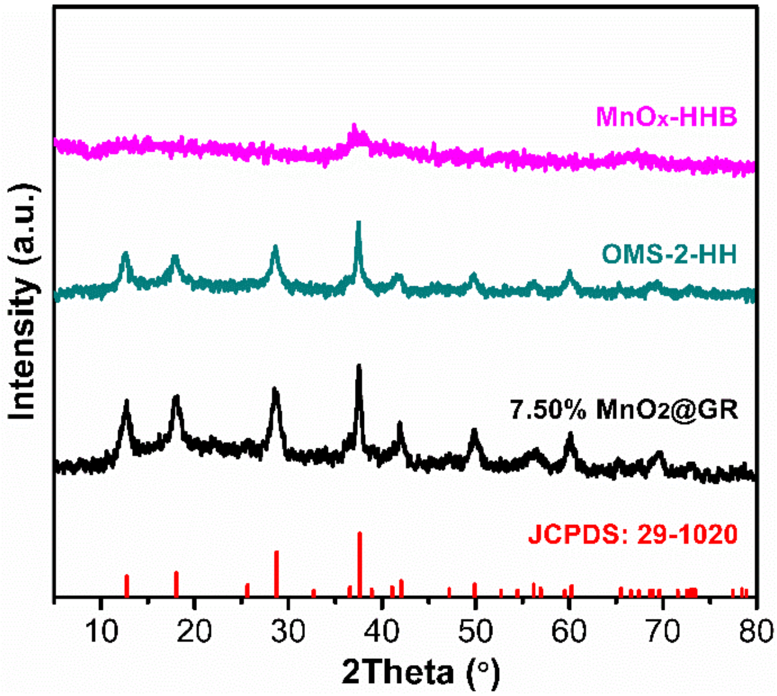

Figure S14. The XRD patterns of OMS-2-HH and MnO_x-HHB catalysts. XRD patterns of 7.50% MnO_2@GR, OMS-2-HH and MnO_x-HHB.

To compare the catalytic performance of 7.50% MnO_2@GR with the reported materials, OMS-2-HH[6] and MnO_x-HHB[7] were prepared. As shown in **figure S14**, OMS-2-HH displayed a pure α-MnO_2 (JCPDS No. 29-1020) and MnO_x-HHB has no obvious diffraction peak, in line with the reported results.

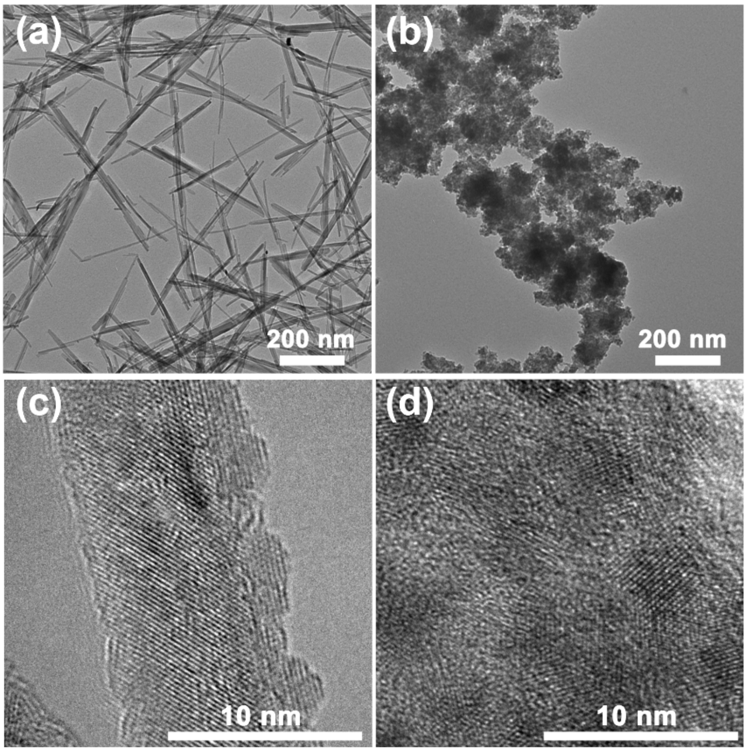

Figure S15. TEM images of OMS-2-HH (a) and MnO_x-HHB (b). HRTEM images of OMS-2-HH (c) and MnO_x-HHB (d).

Figure S15 shown the TEM and HRTEM images of OMS-2-HH and MnO_x-HHB, which are consisted with the the reported results. OMS-2-HH presented a rough surface, which induced abundant surface oxygen vacancy. MnO_x-HHB presented a low crystallinity and abundant crystal boundary formed, which resulted in a low AOS of Mn atoms (the reported AOS of Mn atoms was 3.43).

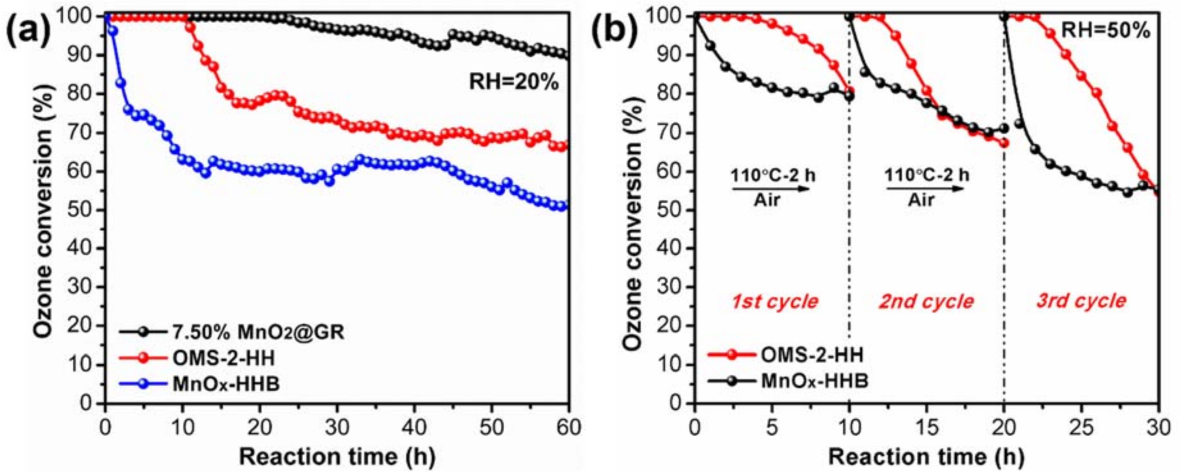

Figure S16. The comparison on the regeneration ability of MnO_2@GR and reported catalysts under different conditions. (a) Comparison of ozone conversion with 7.50% MnO_2@GR and other reported catalysts. (b) Ozone conversion on OMS-2-HH and MnO_x-HH at 50% RH and their regeneration performance (Regenerate condition: 110°C, air atmosphere).

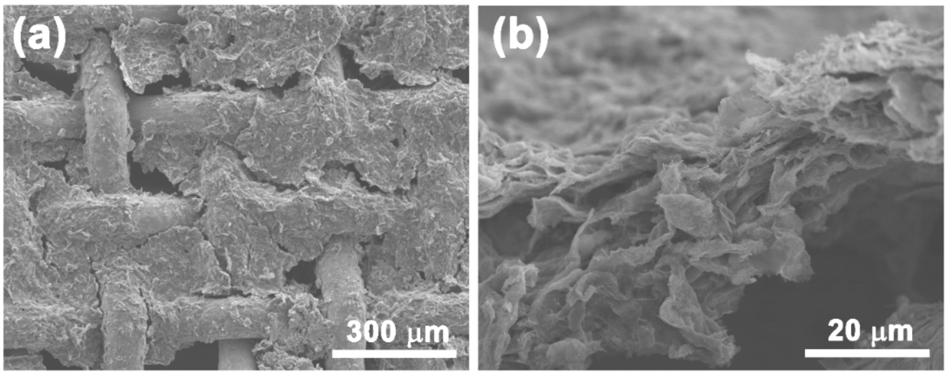

Figure S17. SEM images of 7.50% MnO_2@GR coated stainless steel mesh.

As shown in **figure S17**, 7.50% MnO_2@GR was built into a lamellar structure and uniformly coated on the wire mesh (10×15 cm), using graphene layer as the framework.

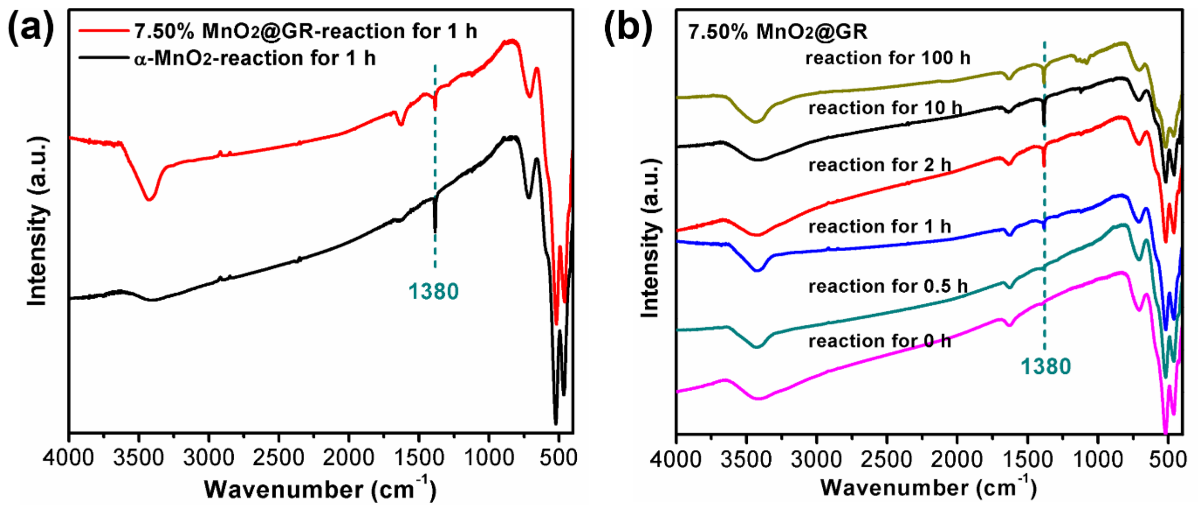

Figure S18. The accumulation of intermediated oxygen species. (a) FT-IR spectra of aMnO$_2$ and 7.50% MnO$_2$@GR treated with O$_3$ for 1 h. (b) FT-IR spectra of 7.50% MnO$_2$@GR treated with O$_3$ for different time.

Figure S18a showed the accumulation of the intermediated oxygen species on the 7.50% MnO$_2$@GR was lower than the α-MnO$_2$ nanowire. **Figure S18** showed the accumulation of the intermediated oxygen species on the 7.50% MnO$_2$@GR was remained stable after 2 h.

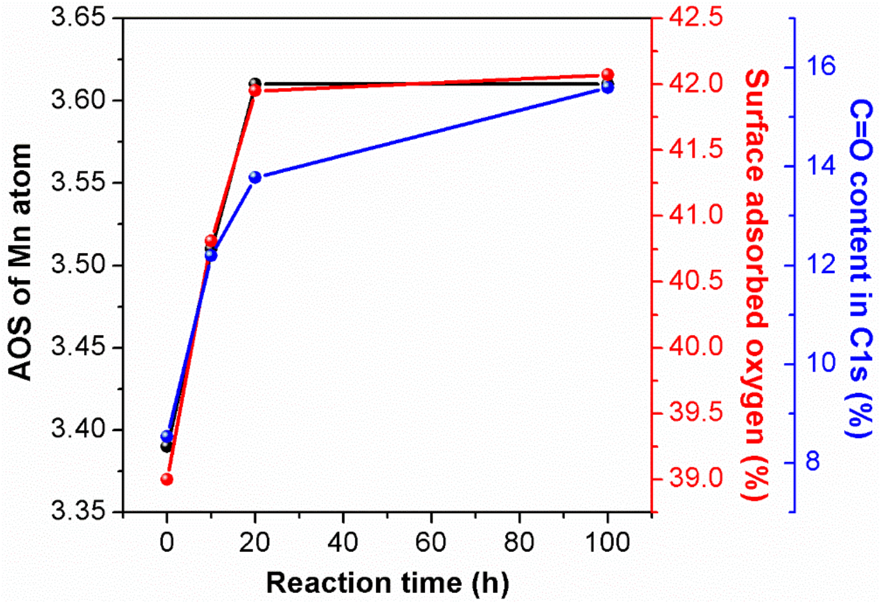

Figure S19. The AOS of Mn, surface adsorbed oxygen content in O 1s and C=O content in C1s after 7.50% MnO_2@GR treated with ozone for different time.

As shown in **Figure S19**, the AOS of Mn and surface adsorbed oxygen content have same variation tendency. The variation of C=O content was slightly slow, which confirmed that the nongraphitic impurities in graphene shells would be oxidized to C=O groups and COOH groups. These results suggested the oxidation state of Mn was closely related to the surface oxygen species in 7.50% MnO_2@GR.

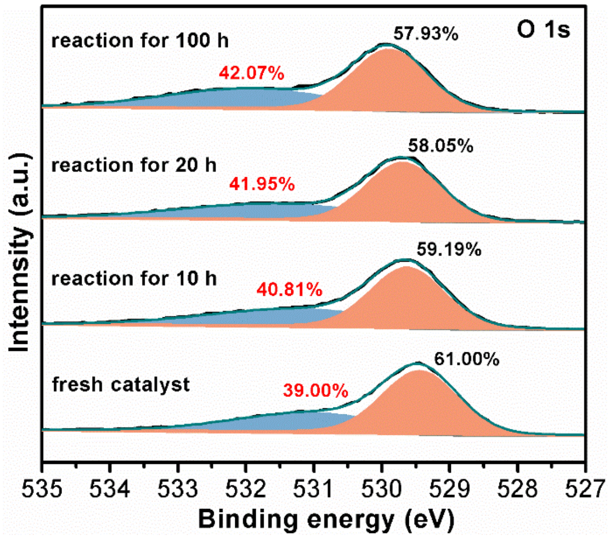

Figure S20. Analysis of the surface bonding environment via XPS. O 1s spectra of 7.50% MnO_2@GR treated with ozone for different time.

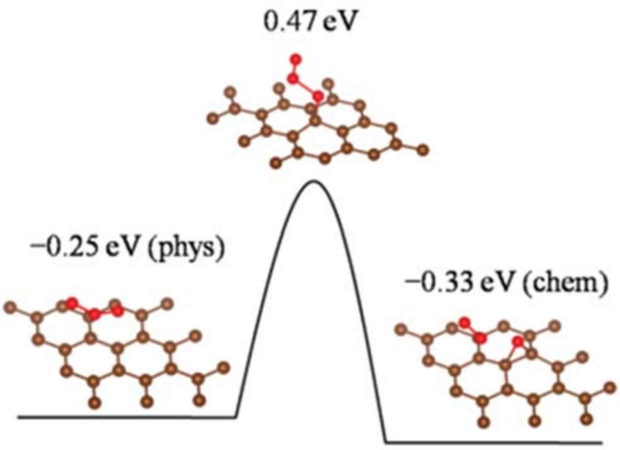

Figure S21. Dissociative chemisorption of an ozone molecule from the physisorbed state is shown with the transition state.[8]

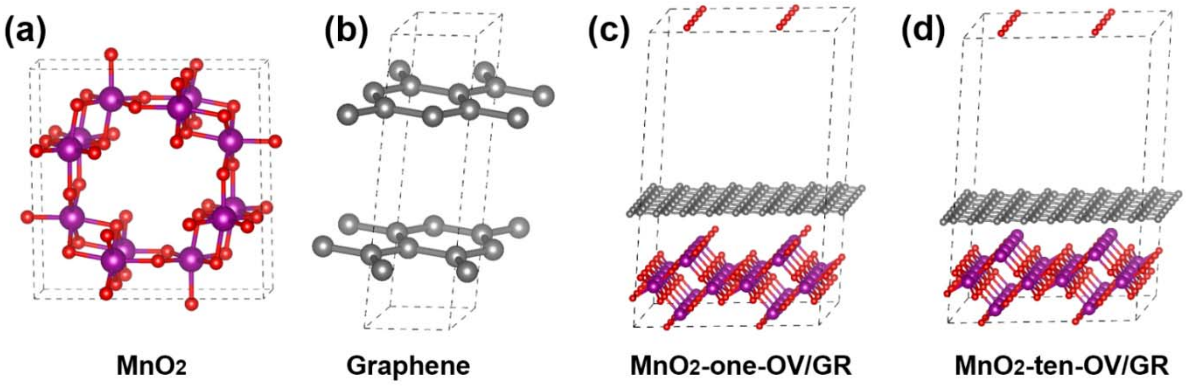

Figure S22. The initial crystal structures of MnO_2 (a) and graphene. (b) The models of MnO_2-one-OV/GR and MnO_2-ten-OV/GR heterojunctions.

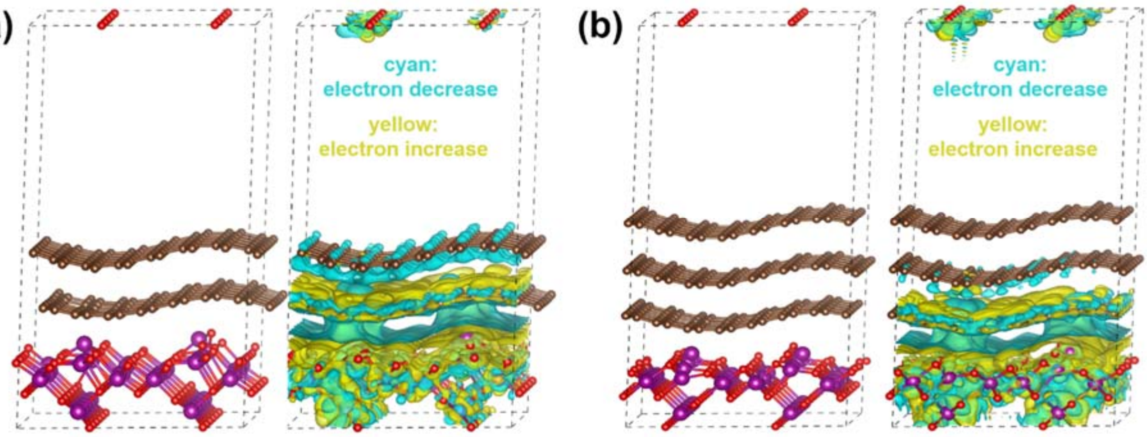

Figure S23. The optimized structure of MnO_2-ten-OV@GR with different graphene layers and their charge density differences. (The yellow and cyan regions refer to increased and decreased charge distributions, respectively. The isosurface value of the colour region is 0.0001e·$Å^{-3}$. The purple, red and gray ball in the models corresponds to the Mn, oxygen and carbon atoms, respectively.)

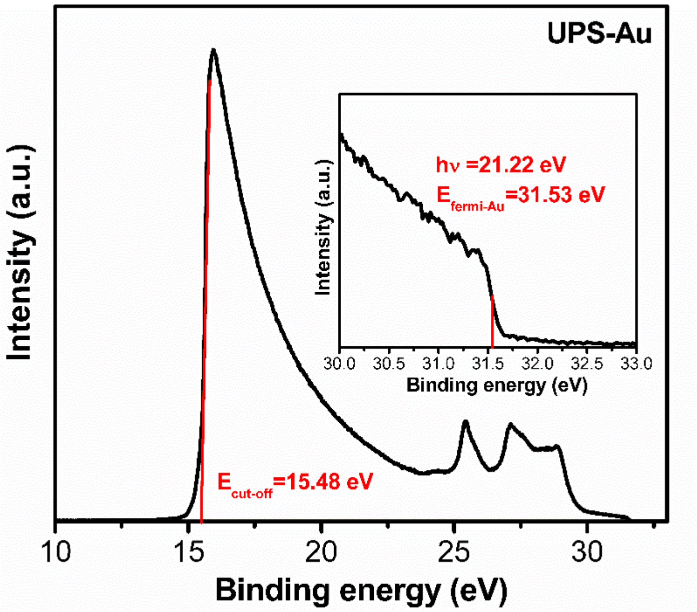

Figure S24. Ultraviolet Photoelectron Spectroscopy of Au.

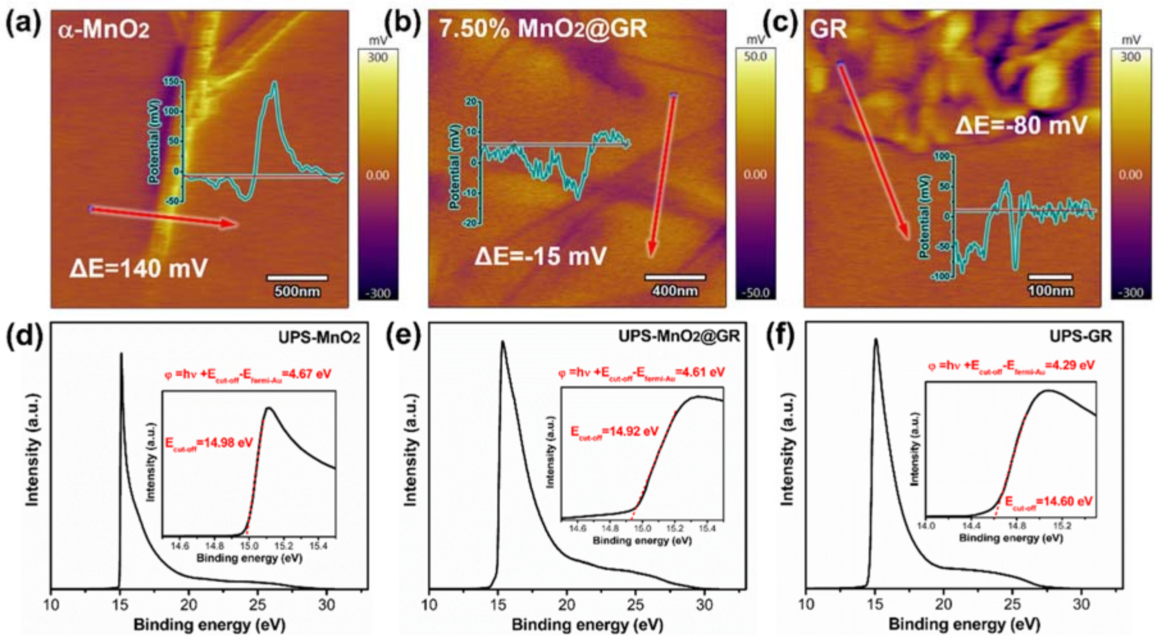

Figure S25. The unique surface electronic structure in MnO_2@GR. Surface potential of α-MnO_2 (a), 7.50% MnO_2@GR (b) and GR (c). UPS spectra of α-MnO_2 (d), 7.50% MnO_2@GR (e) and GR (f).

To explore the reason why the accumulation of peroxide species is inhibited, the surface electronic structure is investigated. Firstly, the average electronic potential on the catalyst surface is measured by Atomic Force Microscope with a Kelvin Probe. As shown in **figure S25a-c**, the surface potential of α-MnO_2 nanowire is higher (140 mV) than that of the mica sheet, while that of GR is lower (80 mV) than that of the mica sheet. For 7.50% MnO_2@GR, the surface potential is lower (15 mV) than that of the mica sheet, between the value of α-MnO_2 nanowire and 7.50% MnO_2@GR. These results indicate that the average work function is varied as followed: α-MnO_2 > 7.50% MnO_2@GR > GR. As a result, the difference on work function drives the electrons transfer from the graphene shells to inner α-MnO_2 nanofiber in the core-shell structure of MnO_2@GR. Besides, the average work function (4.67 eV for α-MnO_2, 4.61 eV for 7.50% MnO_2@GR, and 4.29 eV for GR) calculated from the UPS spectra (**figure S24**) further confirms that the graphene shell indeed modifies the surface electronic structure as shown in **figure S25d-e**.

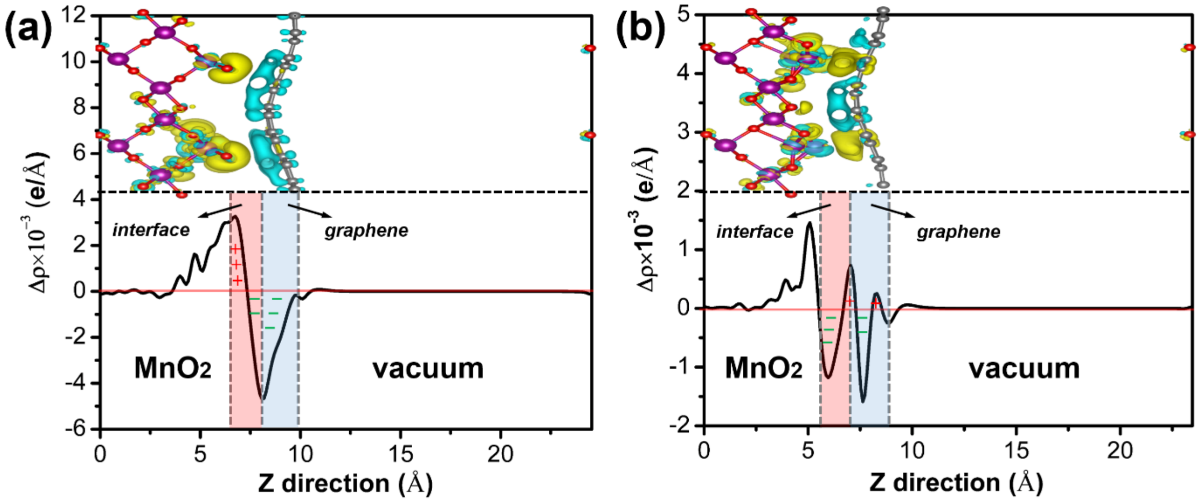

Figure S26. The front view of the charge density differences (above) in MnO_2-one-OV@GR (a) and MnO_2-ten-OV@GR (b). The isosurface value of the color region is 0.01e·Å^{-3}. Planar-averaged electron density difference (below) in MnO_2-one-OV@GR (a) and MnO_2-ten-OV@GR (b) along the Z direction, where the positive values refers to the increased electron and the negative values refers to the decreased charge distribution.

As shown in **figure S26a**, the electron density increased on the surface of MnO_2 and decreased on graphene shell, suggesting the electron transfer from MnO_2 to graphene in MnO_2-one-OV@GR. However, for MnO_2-ten-OV@GR (**figure S26b**), the electron density difference is different and varied with the position, which further confirmed the electron transfer direction depends on the exposed atoms on the surface of MnO_2.

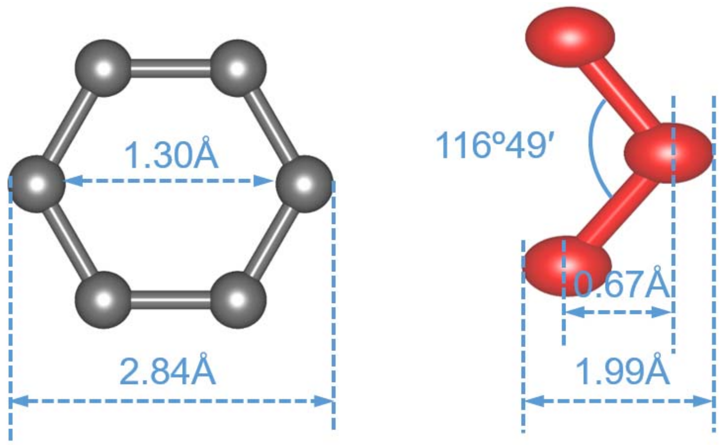

Figure S27. The model of graphene unit (left) and ozone molecule (right).

As shown in **figure S27**, the carbon-carbon bond was 1.42Å in graphene and the covalent radius of carbon atom was 0.77Å. Thus, the biggest pore size of benzene ring was 1.30Å considering the covalent radius. For ozone molecule, the oxygen-oxygen bond was 1.28Å and its angle was 116°49'. The covalent radius of oxygen atom was 0.66Å. Thus, the smallest size was 1.99Å. Thus, ozone molecule can't enter the confined space between graphene layer and manganese oxide by the hole of benzene ring. Although some small molecule can enter the confined space under graphene through open channels at island edges, the process was very slow.[9] However, ozone catalytic decomposition on 7.50% MnO_2@GR was quick and high-throughput. Therefore, the surface of Mn in the confined space between graphene layer and manganese oxide was not the main active site for ozone decomposition.

Table S1. Comparison of ozone catalytic decomposition performance between MnO_2@GR and other reported materials.

Catalyst	Inlet ozone concentration [ppm]	Reaction temperature [℃]	Relative humidity [%]	Space velocity [L/g·h]	Ozone conversion [%]	References
**MnO_2@GR**	**50**	**25**	**20**	**540**	**100 (20 h) / 80 (100 h)**	**This work**
			**50**		**67 (30 h)**	
**MnO_2@GR (coated on stainless steel mesh)**	**50**	**25**	**50**	**216**	**70 (100 h)**	
**OMS-2-HH**	**40**	**30**	**90**	**600**	**75 (6 h)**	**6**
Fe-α-MnO_2	10000	25	90	12	90 (8 h)	10
$LaFeO_3$	200	25	90	240	85 (8 h)	11
Cu_2O Cube	200	25	80	60	100 (8 h)	12
**MnO_x-HHB**	**20**	**25**	**50**	**600**	**100 (12 h)**	**7**
W-α-MnO_2	120	25	65	660	50 (4 h)	13
Ce-α-MnO_2	40	30	65	840	96 (6 h)	14
Ag-MnO_x	40	30	65	840	81 (6 h)	15
Fe-MnO_x	100	25	60	660	73 (6 h)	16
$MnCO_3$+MnO_2	120	25	50	600	65 (5 h)	17
α-MnO_2 nanofiber	23	25	45	880	80 (2 h)	18
Co-MnO_x	1000	25	50	48	66 (12 h)	19
α, β, γ-MnO_2	14		dry	660	-MnO_2 > - MnO_2 > -MnO_2	20
$MnCO_3$	14	25	dry	460	85 (22 h)	21

To define the advantages of MnO_2@GR in ozone conversion, the performance of latest reported catalyst was listed in **Table S1**. Among these materials, OMS-2-HH and MnO_x-HHB displayed best performance for ozone decomposition.

Table S2 Lattice parameters of MnO_2 layer, graphene layer, MnO_2-one-OV/GR and MnO_2-ten-OV/GR heterojunctions.

Model	a (Å)	b (Å)
**MnO_2**	14.618	16.526
**graphene**	14.793	17.258
**MnO_2-one-OV/GR**	14.705	16.893
**MnO_2-ten-OV/GR**	14.705	16.893

References

1. Chen C., Xu K., Ji X., Zhang B., Miao L. & Jiang J. Enhanced electrochemical performance by facile oxygen vacancies from lower valence-state doping for ramsdellite-MnO_2. *J. Mater. Chem. A* **3**, 12461-12467 (2015).
2. Zhu G., et al. Surface oxygen vacancy induced α-MnO_2 nanofiber for highly efficient ozone elimination. *Appl. Catal., B* **209**, 729-737 (2017).
3. Chen S., Zhu J., Wu X., Han Q. & Wang X. Graphene Oxide−MnO_2 Nanocomposites for Supercapacitors. *ACS Nano* **4**, 2822-2830 (2010).
4. Xiao Z., Ning G., Ma X., Zhao L., Yu Y. & Wang H. Hydrothermal assembly of MnO-graphene core-shell nanowires with superior anode performance. *Carbon* **142**, 461-467 (2019).
5. Qiu B., Xing M., Zhang J. Mesoporous TiO_2 Nanocrystals Grown in Situ on Graphene Aerogels for High Photocatalysis and Lithium-Ion Batteries. *J. Am. Chem. Soc.* **136**, 5852-5855 (2014).
6. Ma J., Wang C. & He H. Transition metal doped cryptomelane-type manganese oxide catalysts for ozone decomposition. *Appl. Catal., B* **201**, 503-510 (2017).
7. Liu S., Ji J., Yu Y. & Huang H. Facile synthesis of amorphous mesoporous manganese oxides for efficient catalytic decomposition of ozone. *Catal. Sci. Technol.* **8**, 4264-4273 (2018).
8. Lee, G., Lee, B., Kim, J. & Cho, K. Ozone Adsorption on Graphene: Ab Initio Study and Experimental Validation. *J. Phys. Chem. C* **113**, 14225-14229 (2009).
9. Mu, R. et al. Visualizing Chemical Reactions Confined under Graphene. *Angew. Chem. Int. Ed.* **51**, 4856-4859 (2012).
10. Lian Z., Ma J. & He H. Decomposition of high-level ozone under high humidity over Mn–Fe catalyst: The influence of iron precursors. *Catal. Commun.* **59**, 156-160 (2015).
11. Gong S., Xie Z., Li W., Wu X., Han N. & Chen Y. Highly active and humidity resistive perovskite $LaFeO_3$ based catalysts for efficient ozone decomposition. *Appl. Catal., B* **241**, 578-587 (2019).
12. Gong S., et al. Low temperature decomposition of ozone by facilely synthesized cuprous oxide catalyst. *New J. Chem*. **41**, 4828-4834 (2017).
13. Yang Y., Jia J., Liu Y. & Zhang P. The effect of tungsten doping on the catalytic activity of α-MnO_2 nanomaterial for ozone decomposition under humid condition. *Appl. Catal., A* **562**, 132-141 (2018).
14. Li X., et al. Oxygen Vacancies Induced by Transition Metal Doping in γ-MnO_2 for Highly Efficient Ozone Decomposition. *Environ. Sci. Technol.* **52**, 12685-12696 (2018).

15. Li X., Ma J., Zhang C., Zhang R. & He H. Facile synthesis of Ag-modified manganese oxide for effective catalytic ozone decomposition. *J. Environ. Sci.* **80**, 159-168 (2019).

16. Jia J., Yang W., Zhang P. & Zhang J. Facile synthesis of Fe-modified manganese oxide with high content of oxygen vacancies for efficient airborne ozone destruction. *Appl. Catal., A* **546**, 79-86 (2017).

17. Liu Y., Zhang P., Zhan J. & Liu L. Heat treatment of $MnCO_3$: An easy way to obtain efficient and stable MnO_2 for humid O_3 decomposition. *Appl. Surf. Sci.* **463**, 374-385 (2019).

18. Jia J., Zhang P. & Chen L. The effect of morphology of α-MnO_2 on catalytic decomposition of gaseous ozone. *Catal. Sci. Technol.* **6**, 5841-5847 (2016).

19. Tao L., Zhao G., Chen P., Zhang Z., Liu Y. & Lu Y. High-Performance Co-MnO_x Composite Oxide Catalyst Structured onto Al-Fiber Felt for High-Throughput O_3 Decomposition. *ChemCatChem* **11**, 1131-1142 (2019).

20. Jia J., Zhang P. & Chen L. Catalytic decomposition of gaseous ozone over manganese dioxides with different crystal structures. *Appl. Catal., B* **189**, 210-218 (2016).

21. Jia J. & Zhang P. Catalytic Decomposition of Airborne Ozone by $MnCO_3$ and its Mechanism. *Ozone: Sc. Eng.* **40**, 21-28 (2018).
